# Vegetated landscape coherence and complexity shape psychophysiological stress recovery in campus courtyards

**DOI:** 10.1371/journal.pone.0347162

**Published:** 2026-04-27

**Authors:** Yaling Gao, Qunyue Liu, Wenxin Yang, Lingfang Lin, He Huang

**Affiliations:** 1 School of Design, Fujian University of Technology, Fuzhou, China; 2 Department of Forest Resources Management, Faculty of Forestry, University of British Columbia, Vancouver, British Columbia, Canada; 3 College of Architecture and Urban Planning, Fujian University of Technology, Fuzhou, China; 4 College of Landscape Architecture and Art, Fujian Agriculture and Forestry University, Fuzhou, China; 5 National Center for Water Conservancy Scenic Area Research, Fuzhou, China; Macau University of Science and Technology, MACAO

## Abstract

University students across urban campuses face sustained stress loads, making everyday restorative opportunities paramount. We ran a randomized virtual-reality experiment (N = 282) crossing landscape coherence (low, moderate, high) and complexity (low, moderate, high) in teaching-building courtyards, plus a non-vegetated control, to interrogate how designable visual attributes govern recovery. Complexity was parameterized with Shannon entropy while coherence reflected planting order. Heart rate, RMSSD, skin conductance level (SCL), and the Perceived Restorativeness Scale (PRS) were collected across baseline, stress, and recovery phases. Vegetated scenes improved SCL and PRS relative to the control. PRS rose monotonically with complexity and peaked at moderate-to-high coherence. Crucially, coherence moderated SCL responses: biodiversity-rich scenes only delivered autonomic settling when ordered, revealing a conditional mechanism that ties information-processing theory to stress-reduction pathways. The entropy-guided framework therefore refines prevailing restorative theories and yields a portable VR protocol that international teams can adapt with local species palettes. The findings translate into globally relevant guidance for campus and urban designers seeking micro-restorative infrastructure in dense settings.

## 1 Introduction

University students worldwide face sustained mental-health burdens that exceed those of the general population. Multinational WMH-ICS surveys consistently report high stress loads and impaired functioning among first-year cohorts [1], and a meta-analysis of more than 93,000 Chinese students estimates a 34.7% depression prevalence, over five times the national adult rate [2]. Complementary evidence from Europe and North America indicates rising anxiety, sleep disruption, and burnout across campus populations, patterns tightly coupled with pandemic recovery, economic uncertainty, and intensified academic competition [3,4]. Beyond individual risk profiles, population-level studies demonstrate that everyday access to nearby nature systematically shapes mental-health outcomes, with ambient greenness and accessible blue/green spaces linked to lower psychological distress and narrower inequalities [4–6]. Designing campus environments that intentionally cultivate well-being is therefore a global imperative rather than a geographically bounded issue.

As urban universities densify and vertical campuses proliferate, the spatial interstices between teaching buildings, dormitories, and student services have emerged as everyday “micro-restorative” arenas [7,8]. Courtyards, linear greens, and rooftop terraces double as transit corridors, informal classrooms, and social refuges, yet their design is frequently governed by circulation efficiency or branding rather than health evidence. International case studies—from Canadian living labs to European biophilic retrofits—show that modest alterations in vegetation layering, shade, and soundscapes can measurably influence cognitive performance and stress physiology during short breaks [3,9,10]. These findings highlight the need for transferable design knowledge that helps campus and urban designers orchestrate restorative affordances within compact, high-demand spaces.

Two foundational theories articulate why contact with urban nature supports recovery across cultures. Stress Reduction Theory (SRT) holds that unthreatening natural settings dampen sympathetic arousal and facilitate parasympathetic rebound [11–14], whereas Attention Restoration Theory (ART) posits that environments rich in fascination and extent replenish depleted directed attention [15]. Decades of field and laboratory research demonstrate these mechanisms in action: brief visits to urban parks lift mood and subjective vitality [16], and controlled laboratory studies report faster autonomic recovery when stressed participants view nature imagery rather than built scenes [17]. Instruments such as the Perceived Restorativeness Scale (PRS) operationalise the ART/SRT constructs with strong psychometric performance across languages and cultures [18], enabling comparative assessments from Nordic forests to Southeast Asian campuses [3,5].

Yet a universal dictum that “green is good” masks critical variation. Landscape perception research shows that visual organisation and biodiversity influence preference, restoration, and behavioural outcomes, often in non-linear ways. Building on Kaplan’s information-processing perspective, coherence reflects the clarity with which observers can grasp spatial structure, whereas complexity indexes the richness and variety of elements that invite exploration [19–21]. Early experimental psychologists theorised inverted-U relationships between complexity and preference [22–24], while contemporary eye-tracking and photogrammetric studies frequently report near-linear gains in perceived restorativeness as visual richness rises, provided that scenes remain legible [25,26]. Parallel work on “landscape design intensity” demonstrates that multi-layered planting and articulated edges can heighten fascination but may also induce cognitive overload when ordering cues are absent [27,28]. These mixed findings suggest that coherence and complexity act jointly—not independently—to translate information-rich vegetation into restorative experience.

Despite decades of restorative research, experiments that manipulate coherence and complexity jointly remain scarce, and theoretical treatments often assume additive rather than conditional effects. Many foundational studies relied on two-dimensional photographs, which are vulnerable to framing, familiarity, and lighting confounds and provide limited traction on psychophysiological mechanisms beyond self-report [29,30]. Others sampled narrow bands of biodiversity or legibility, leaving the potential interaction under-specified [28,31]. Cross-cultural coverage is likewise uneven: most psychophysiological work has been conducted in Western contexts, even though the densification trajectories and vegetation palettes of Asian, African, and Latin American campuses differ markedly [7,8]. Consequently, designers lack causal evidence about when richer planting palettes advance or hinder recovery, and theorists lack an integrated model that reconciles attentional fascination with the need for spatial predictability.

We address these gaps by advancing an entropy-informed coherence–complexity framework. Shannon entropy quantifies planting diversity in bits, enabling explicit calibration of species richness and proportional balance, while coherence is operationalised through clustering regularity, sight-line orchestration, and thematic repetition. Conceptually, the framework proposes a two-stage restorative mechanism: biodiversity supplies the informational richness that fuels fascination and micro-restorative engagement, and coherence moderates whether that richness is interpreted as inviting exploration or as disorder that perpetuates stress. Framing coherence as a moderator links Kaplan’s information-processing account with SRT by positing that legibility is the gateway through which complexity can trigger parasympathetic rebound rather than sustained vigilance. Because entropy and coherence can be computed or scripted within design software, the framework also paves the way for reproducible comparisons across climates, cultures, and planting palettes. Within evidence-based design practice, these metrics can anchor parametric workflows and post-occupancy audits, turning theoretical constructs into quantitative targets for campus retrofit and new-build projects worldwide.

Accordingly, the study delivers three contributions. First, it moves beyond generic nature–built contrasts to estimate the independent influence of coherence and complexity—attributes that practitioners can intentionally manipulate—on student stress recovery. Second, it theorises and tests the moderating role of coherence on biodiversity, revealing when rich planting palettes actually convert to autonomic benefits and thereby refining theories of restorative experience. Third, it deploys an immersive VR protocol with objective physiology (HR, RMSSD, SCL) and validated psychological measures (PRS) to produce a portable evaluation method that international campus and urban designers can adapt to local species mixes. By situating the experiment in teaching-building courtyards—prototypical micro-restorative spaces in dense universities worldwide—we contribute evidence that is immediately transferable to comparable institutions navigating rapid urbanisation. In doing so, the study extends the global knowledge base on how specific visual attributes can be tuned to support student well-being, offering a common language for planners, landscape architects, and health researchers.

We therefore propose the following hypotheses:

H1: Exposure to vegetated courtyard scenes will lead to greater stress recovery compared to the non-vegetated control scene.
H2: Landscape coherence will be positively associated with stress recovery, with higher coherence leading to greater restorative benefits.
H3: Landscape complexity will be positively associated with stress recovery, particularly for psychological restoration.
H4: Coherence and complexity will interactively influence the restorative outcomes of participants’ psychological and physiological indicators.

## 2 Methods

### 2.1 Experimental design

This study utilized a two-factor, between-subjects randomized controlled experiment, with Complexity (low, moderate, high) and Coherence (low, moderate, high) as independent variables. The dependent variable was the recovery effect, defined as the mean during the 5-min recovery phase minus the mean during the 5-min stress phase for each physiological outcome. Physiological indicators were continuously monitored across phases, and the Perceived Restorativeness Scale (PRS) was administered at the end to examine stress recovery under varying levels of landscape coherence and complexity, as well as their interaction effects.

### 2.2 Participants

To determine sample size, we used G*Power 3.1.9.7 (ANOVA, fixed effects, omnibus, one-way), setting effect size at 0.3 and α at 0.05; the analysis indicated N = 280 for 0.95 power. We recruited N = 282 physically healthy undergraduate, master’s, and doctoral students from Fujian University of Technology (FUT) via WeChat announcements and posters. Participants arrived individually at the laboratory according to scheduled appointments in order to minimize prolonged waiting before the experiment. Before the formal experiment, a staged screening procedure was implemented. The recruitment notice excluded students with obvious medical histories (e.g., cardiovascular disease, heart disease, color blindness, or epilepsy) or poor recent physical condition, and required participants to avoid behavioral habits that might interfere with the experimental results. Upon arrival, during the in-formed-consent and preparation/waiting stages, participants were re-asked about their recent physical condition, anxiety status, and relevant lifestyle habits (e.g., staying up late, alcohol consumption, and coffee intake), and underwent pre-test assessments of heart rate and blood pressure. To reduce heterogeneity in participants’ baseline psychophysiological status, only those who met the screening criteria proceeded to instrumentation and the formal experimental procedure. As compensation, participants received educational re-sources. The cohort comprised 161 females and 121 males (mean age 25.4 ± 5.0). Detailed sociodemographic data by arm are shown in Table 1. The study followed FUT ethics guidelines and was approved by the university ethics committee. All participants gave informed consent; the questionnaire was described and completed in Chinese. Participants were randomized to one of the ten arms using a computer-generated simple random sequence (participants were randomly assigned and were unaware of the assigned scene).

**Table 1:** Descriptive statistics of socio-demographic characteristics.

Scenario	headcount	Gender	Age (mean, SD)
CG	27	Female 15	24.1,4.2
		Male 12	
A1	29	Female 14	25.4, 3.3
		Male 15	
A2	28	Female 14	26.5, 3.6
		Male 14	
A3	28	Female 16	26.2, 3.3
		Male 12	
B1	29	Female 16	25.4, 3.1
		Male 13	
B2	28	Female 16	26.1, 3.8
		Male 12	
B3	27	Female 15	24.8, 4.2
		Male 12	
C1	28	Female 12	25.5, 3.9
		Male 16	
C2	29	Female 17	27.9, 3.2
		Male 12	
C3	29	Female 16	22.6, 4.4
		Male 13	

### 2.3 Experimental stimuli and operationalizations

To examine responses to landscape coherence and complexity in campus courtyards, we designed nine 3D virtual environments (3 × 3 Coherence × Complexity) using AutoCAD 2019, SketchUp 2018, and Enscape 2.4, plus one non-vegetated built courtyard control. The nine vegetated scenes varied only in vegetation (trees, shrubs, grasses); built elements (buildings, roads, plazas, and other hardscape) were held constant in quantities, forms, materials, colors, and volumes. In the digital model development phase, we surveyed courtyard configurations at >120 universities across North, East, and South China, extracting typical patterns (e.g., three-sided courtyards formed by two teaching buildings joined by a corridor, with pavilions, plantings, decorations, walkways). Based on these patterns and our experimental goals, we built in AutoCAD 2019, modeled in SketchUp 2018, and rendered in Enscape 2.4.

#### 2.3.1 Coherence.

**Fig 1:**
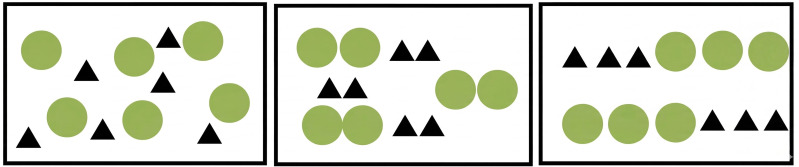
Coherence design diagram.

In the coherence design, the number of plant clusters was the representative metric. Plants were organized in rows, columns, or clusters with similar themes or textures to achieve a structured, orderly visual output. Increasing coherence was realized by reducing the number of clusters. A schematic of the coherence manipulation is shown in Fig 1. Coherence was segmented into three tiers—low, medium, and high—according to spatial arrangement.

#### 2.3.2 Complexity quantified by entropy.

**Fig 2:**
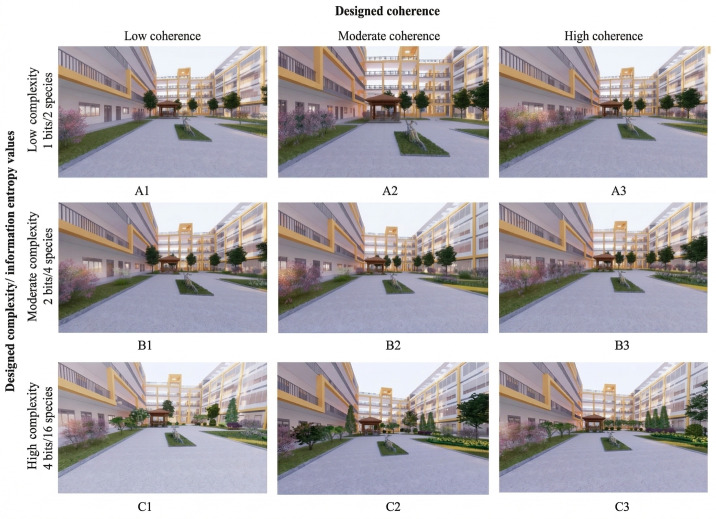
Complexity and coherence virtual scene (Created by the authors using Enscape software).

Guided by Stamps [32] and Kuper [31], we operationalized complexity using information entropy values of 1, 2, and 4 bits (low, moderate, high). The Shannon formulation was:


$$H_{factor}=-\sum_{i=\text{1}}^{nlevels}p_{i}{log}_{\text{2}}p_{i}$$ (1)

where $p_{i}$ is the proportion of vegetation elements in category *i* present in view (e.g., species groups or strata), *n* is the number of categories, and *H* is expressed in bits. By construction, equal abundances of *k* categories yield $H={log}_{\text{2}}k$ (e.g., 2 categories yield 1 bit, 4 categories yield 2 bits, 16 categories yield 4 bits). We targeted three entropy levels—1, 2, and 4 bits—corresponding to low, moderate, and high complexity, respectively, both to guide scene construction and to verify post-hoc that the nine vegetated scenes spanned the intended range. Table 2 presents the mapping between entropy levels (1, 2, 4 bits), plant types, and plant counts. Low complexity (1 bit) used two species totaling 48 plants (24 + 24); moderate complexity (2 bits) used four species totaling 48 plants (12 × 4); high complexity (4 bits) used sixteen species totaling 48 plants (3 × 16). Each scene included at least 12 trees; the shrub/herbaceous composition varied by complexity level. All non-vegetation features (lighting, sky, color palette, and hardscape) were held constant across scenes. The non-vegetated built courtyard control was generated from the same base model by removing all vegetation while keeping the built and hardscape elements identical. The 3 × 3 matrix of the nine vegetated Coherence × Complexity scenes is shown in Fig 2.

**Table 2:** Combination of information entropy values with landscape complexity and corresponding plant types and quantities (scene-level totals fixed at 48 plants for all three complexity levels).

Complexity (information entropy value)	Entropy value	Plant Types	Quantity
High (4 bit)	Trees (4 Types)	Deciduous Trees	3
		Deciduous Trees	3
		Evergreen Trees	3
		Evergreen Trees	3
	Shrubs（6 Types）	Deciduous Shrubs	3
		Deciduous Shrubs	3
		Flowering Shrubs	3
		Flowering Shrubs	3
		Evergreen Shrubs	3
		Evergreen Shrubs	3
	Grass (6 Types)	Ornamental Grasses	3
		Ornamental Grasses	3
		Flowering herbaceous	3
		Flowering herbaceous	3
		Colorful Foliage herbaceous	3
		Colorful Foliage Herbaceous	3
Moderate (2 bit)	Trees (1 Type)	Deciduous Trees	12
	Shrubs (1 Type)	Flowering Shrubs	12
	Grass (2 Types)	Ornamental Grasses	12
		Flowering herbaceous	12
Low (1 bit)	Trees (1 Type)	Deciduous Trees	24
	Shrubs (1 Type)	Flowering herbaceous	24

### 2.4 Apparatus and data acquisition

VR presentation used HTC VIVE Pro. Physiological data were recorded with an Ergo-LAB Physiological Multi-Gauge: photoplethysmography (PPG, earlobe) for HR/HRV and electrodermal activity (EDA) via Ag/AgCl electrodes on the index and middle fingers; signals were streamed to a host PC via Bluetooth. Participants were seated indoors under stable lighting. RMSSD served as the primary time-domain HRV index suited to short-term (~5-min) windows; SCL was treated as tonic EDA with standard site preparation and artifact screening. Given that PPG-derived HRV aligns best with ECG during seated rest, HRV estimates during stress were interpreted cautiously.

### 2.5 Procedure and stress induction

**Fig 3:**
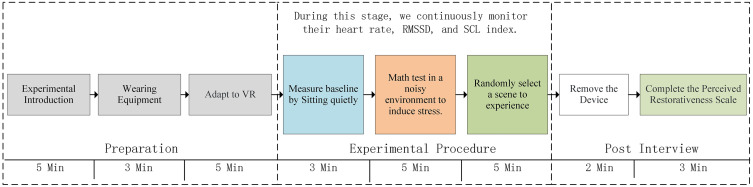
Timeline of experimental procedure and sampling (one visit).

Participants wore an HTC VIVE Pro headset to enter the VR environment. After consent, briefing, and instrumentation, they completed a 5-min indoor rest and VR familiarization, followed by a 5-min seated baseline recording. This standardized pre-test stage was intended to reduce potential interference associated with waiting time and equipment novelty and to stabilize baseline status before the formal task. Stress was induced with a 5-min task block (2-min memory + 3-min mental arithmetic) presented under mixed noise (traffic, machinery, student–teacher interaction; ~ 70 dB LAeq), following commonly used laboratory approaches for eliciting acute psychological stress by combining cognitive load with environmental disturbance [33]. Immediately afterward, participants viewed a 5-min recovery scene—randomly assigned to one of nine vegetated scenes or the non-vegetated built courtyard control. This short recovery exposure was grounded in SRT and ART and informed by prior studies showing psychophysiological recovery after exposure to natural or virtual-natural environments [17,34,35]. The manipulations of coherence and complexity followed Kaplan’s information-processing framework, with complexity operationalized using Shannon entropy as guided by Kaplan [15,36], Stamps [27,32], Stamps III [37], and Kuper [31]. After VR exposure, the PRS was administered. All testing occurred in the Ergonomics Laboratory of FUT (September 1 to October 30, 2024) under air-conditioned indoor conditions (temperature set to ~26°C). The procedure and timings are illustrated in Fig 3.

### 2.6 Outcome measures

#### 2.6.1 Psychophysiology.

HR (bpm), RMSSD (ms), and SCL (µS) were recorded continuously across baseline (5 min), stress (5 min), and recovery (5 min).

#### 2.6.2 Psychological restoration.

Following the recovery exposure, participants’ psychological restoration was assessed with the Perceived Restorativeness Scale (PRS). Grounded in Attention Restoration Theory (ART), the PRS is a widely used instrument for evaluating the restorative quality of environments and associated psychological stress responses [9,27]. To minimize participant burden, we employed the short, culturally adapted Chinese version developed by Wang et al. [38]. This version demonstrates good internal consistency in previous studies (Cronbach’s α = 0.801) and has been widely applied in Chinese studies of stress restoration. In the present sample, after reverse-coding the negatively worded item, the PRS showed good internal consistency (Cronbach’s α = 0.833). The instrument operationalizes the four canonical dimensions of restorative environments articulated by Kaplan and Peterson [39]—Being Away, Fascination, Extent, and Compatibility. Items were rated on a 7-point Likert scale (1 = strongly disagree, 7 = strongly agree), and the questionnaire was administered in Chinese.

### 2.7 Statistical analysis

All analyses were conducted in IBM SPSS Statistics (v27) with α=0.05 (two-tailed). For each outcome (ΔHR, ΔRMSSD, ΔSCL, PRS), hypotheses were tested as follows:

(1) Across 10 conditions (9 vegetated + non-vegetated built control): one-way ANOVA with Bonferroni post-hoc comparisons focused on differences vs the control.
(2) Main effects of Coherence and Complexity (3 levels each): separate one-way ANOVAs for Coherence and for Complexity; Bonferroni post-hoc tests where applicable.
(3) Interaction: two-way ANOVA (Coherence × Complexity) for each outcome; where significant, simple-effects analyses were conducted as reported in the Results.

We report F, p, partial η², and 95% confidence intervals; model assumptions (normality, homoscedasticity) were checked.

## 3 Results

**Fig 4:**
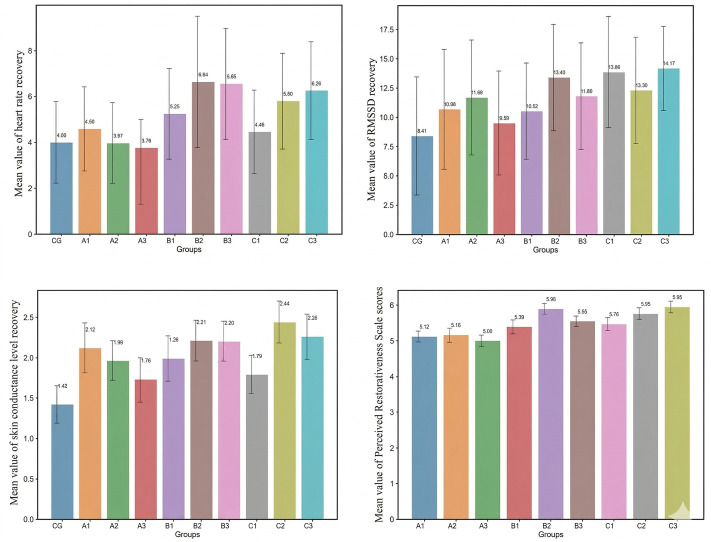
The mean recovery values of physiological and psychological indicators for the ten.

We report findings in the order of the pre-specified hypotheses. Descriptive statistics by condition are shown in Fig 4; summary ANOVAs appear in Tables 3–9.

**Table 3:** Presents the results of an ANOVA that examines the recovery values of physiological and psychological indicators across different groups.

Parameter	Heart Rate	RMSSD	SCL	PRS
*P* Value	0.18	0.70	<0.01*	<0.01*
*F*	1.42	0.71	5.45	10.59
*η2*	0.07	0.02	0.15	0.26
*Df*	9	9	9	9

**Note:** * indicates a statistically significant difference at the 0.01 level (p < 0.01).

**Table 4:** The P-value matrix for the post-hoc analysis of ANOVA conducted for the pairwise comparisons in the SCL analysis.

I	J	Coherence			
		*MD*(I-J)	*SE*	*P*	*95% CI*
CG	A1	0.70	0.18	0.07	0.10 to 1.30
	A2	0.54	0.18	0.17	−0.07 to 1.14
	A3	0.30	0.18	1	−0.30 to 0.91
	B1	0.57	0.18	0.09	−0.03 to 1.17
	B2	0.79	0.18	<0.01*	0.18 to 1.39
	B3	0.78	0.19	<0.01*	0.17 to 1.39
	C1	0.37	0.18	1.00	−0.24 to 0.98
	C2	1.02	0.18	<0.01*	0.42 to 1.62
	C3	0.84	0.18	<0.01*	0.24 to 1.44

I represents the control group, while J represents the nine distinct courtyard landscape groups.* indicates a statistically significant difference at the 0.01 level.

**Table 5:** Descriptive statistics of recovery capabilities are presented for three levels of coherence and complexity: low, moderate, and high.

Variables	statistic	Coherence			Complexity		
		Low	Moderate	High	Low	Moderate	High
HR	*M*	−4.80	−5.37	−5.56	−3.82	−6.34	−5.56
	*SD*	5.75	7.46	6.71	4.79	7.46	7.22
	*CI (95%)*	−3.57,-6.03	−3.76,-6.98	−4.10,-7.01	−2.79,-4.86	−4.72,-7.96	−4.02,-7.11
RMSSD	*M*	11.66	12.46	11,85	10.62	11.89	13.44
	*SD*	12.13	11.97	10.83	12.41	11.24	11.14
	*CI (95%)*	9.06,14.26	9.88,15.04	9.50,14.20	7.94,13.30	9.45,14.33	11.05,15.83
SCL	*M*	−1.97	−2.21	−2.06	−1.94	−2.13	−2.17
	*SD*	0.73	0.68	0.72	0.73	0.67	0.73
	*CI(95%)*	−1.84,-2.12	−2.06,-2.35	−1.91,-2.22	−1.78,-2.09	−1.99,-2.28	−2.01,-2.33
PRS	*M*	5.00	5.41	5.40	4.63	5.43	5.74
	*SD*	0.99	0.98	1.14	0.82	1.09	0.91
	*CI (95%)*	4.79,5.22	5.20,5.62	5.15,5.64	4.46,4.81	5.19,5.66	5.55,5.94

Note that in the table, “M” represents the mean difference between recovery period values and post-pressure values for HR, RMSSD, and SCL. “SD” stands for standard deviation, and “CI” denotes confidence interval.

**Table 6:** Displays the outcomes of ANOVA for the physiological and psychological recovery values of participants across the three levels of coherence scenes.

Parameter	Heart Rate	RMSSD	SCL	PRS
*P* Value	0.74	0.29	0.09	<0.01*
*F*	0.30	1.26	2.46	6.33
*η2*	0.00	0.01	0.02	0.05
*Df*	2	2	2	2

Note: * indicates a statistically significant difference at the 0.01 level.

**Table 7:** Displays the outcomes of ANOVA for the physiological and psychological recovery values of participants across the three levels of complexity scenes.

Parameter	Heart Rate	RMSSD	SCL	PRS
*P* Value	0.10	0.07	0.08	<0.01*
*F*	2.03	2.69	2.62	44.39
*η* ^*2*^	0.03	0.02	0.02	0.26
*Df*	2	2	2	2

Note: * indicates a statistically significant difference at the 0.01 level.

**Table 8:** Post-hoc comparisons of landscape coherence and complexity between different types of setting.

I	J	Coherence				Complexity			
		*MD*(I-J)	*MD*(I-J)	*P*	*95% CI*	*MD*(I-J)	*MD*(I-J)	*P*	*95% CI*
L	M	0.19	0.11	<0.01	−0.45 to 0.12	−0.52	0.07	<0.01	−0.68 to 0.35
	H	−0.18	0.068	0.03	0.34 to 0.02	−0.64	0.07	<0.01	−0.80 to −0.47
M	H	0.11	0.068	0.35	−0.56 to 0.27	−0.12	0.06	<0.01	−0.28 to 0.04

I and J represent three levels of coherence or complexity in the environment, namely low, moderate, and high.

In the table, L denotes Low, M stands for Moderate, and H represents High. * indicates a statistically significant difference at the 0.01 level.

**Table 9:** The results of intergroup main effects analysis on four physiological and psychological indicators were examined through a general linear model.

Variables	*Sum of Class III*	*Df*	*F*	*Sig.*	*η* ^*2*^
HR	387.05	8	1.23	>0.05	0.03
RMSSD	583.60	8	0.53	>0.05	0.02
SCL	12.21	8	3.20	<0.05*	0.09
PRS	74.03	8	10.95	<0.05*	0.26

### 3.1 Vegetated courtyards enhance stress recovery (H1)

Across the ten conditions (nine vegetated designs + non-vegetated built courtyard control), SCL and PRS differed significantly, whereas HR and RMSSD did not (Table 3: SCL, F = 5.45, p < .01, η² = .15; PRS, F = 10.59, p < .01, η² = .26; HR, p = .18; RMSSD, p = .70). Bonferroni post-hoc comparisons against the control (Table 4) indicated that most vegetated scenes yielded larger SCL recovery than the control (exceptions: A3, C1). For illustration, the C2 scene (moderate coherence, high complexity) showed the largest SCL recovery magnitude (|ΔSCL| = 2.44 μS, 95% CI: 2.18–2.70), whereas the control showed the smallest (|ΔSCL| = 1.42 μS, 95% CI: 1.19–1.65). Taken together, H1 is supported for SCL and PRS, but not for HR or RMSSD.

### 3.2 Main effects of coherence and complexity on stress recovery (H2 & H3)

**Fig 5:**
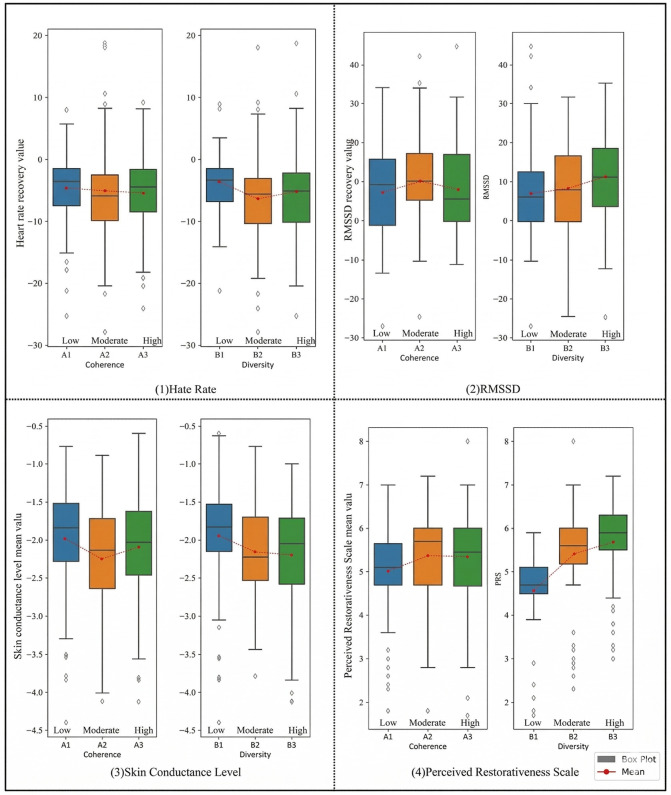
Box plots depicting the recovery values of physiological and psychological indicators among subjects in landscapes characterized by coherence and complexity.

Table 5 presents the descriptive statistics of HR, RMSSD, SCL, and PRS across low, medium, and high levels of coherence and complexity. Fig 5 displays the box plots of these indicators at these levels. As per Table 5 and Fig 5, college students exhibited the best HR recovery at high coherence and medium complexity levels. Specifically, HR decreased by an average of 5.56 bpm (95% CI: 4.10–7.01) at high coherence, followed by medium and low coherence levels. For medium complexity, HR reduced by an average of 6.34 bpm (95% CI: 4.72–7.96), with recovery effects being better at high complexity than at low complexity.

Regarding the RMSSD metric for college students, the best recovery was observed at the medium coherence level, registering a recovery of 12.46 ms (95% CI: 9.88–15.04), followed by high and low coherence levels. In terms of complexity, high complexity showed the best recovery for RMSSD with a value of 13.44 ms (95% CI: 11.05–15.83). However, the box plot (Fig 5) suggests that the recovery effects across the three coherence levels are quite comparable. For the SCL metric, medium coherence level showcased optimal recovery at 2.21μS (95% CI: 2.06–2.35), slightly outperforming high coherence, which in turn was better than low coherence. In the context of complexity, the high level demonstrated the best recovery at 2.17μS (95% CI: 2.01–2.33), followed by medium and low complexity levels. For the psychological evaluation PRS scores, both coherence and complexity showed a similar trend, indicating medium level as the most favorable for recovery, succeeded by high level and least effective being the low level.

To investigate the impact of coherence and complexity on the physiological and psychological recovery of university students, we conducted one-way analysis of variance (ANOVA). This analysis explored the independent effects of coherence and complexity across three levels: low, moderate, and high. The results of the ANOVA for the four indicators (HR, RMSSD, SCL, and PRS) are summarized in Tables 6 and 7. The differences in HR, RMSSD, and SCL were not significant (P > 0.05), suggesting no notable variations in recovery values across different coherence and complexity levels. However, the psychological indicator PRS exhibited significant differences across varying levels of coherence (F = 6.33, P < 0.05, η² = 0.05) and complexity (F = 44.39, P < 0.05, η² = 0.26). Post-hoc tests for the PRS indicator, detailed in Table 8, revealed that low coherence levels significantly differed from moderate and high levels (P < 0.05), whereas no significant difference was observed between moderate and high coherence levels (P > 0.05). In terms of complexity, pairwise comparisons indicated that perceived recovery were significantly lower in environments with low complexity compared to moderate and high levels (P < 0.001). Additionally, perceived recovery were significantly lower in environments with moderate complexity compared to high complexity (P < 0.05). These findings underscore the significant influence of both three-level coherence and three-level complexity on perceived stress recovery among participants. Notably, participants demonstrated optimal perceived stress recovery in environments with moderate coherence, followed by high coherence and then low coherence environments. Furthermore, increasing environmental complexity corresponded to enhanced perceived recovery among participants.

By documenting this conditional pathway, we refine Kaplan’s information-processing model and integrate it with Stress Reduction Theory: informational richness appears to yield restorative fascination only when scene legibility keeps cognitive load within manageable limits. This finding challenges linear interpretations of “more diversity equals more restoration” and instead proposes a two-stage mechanism in which coherence governs whether complexity translates into parasympathetic rebound. Because the entropy and coherence metrics are transparent and transferable, the framework can be stress-tested across climates, cultures, and campus typologies, enabling a concerted international research agenda on micro-restorative design.

### 3.3 Interaction effects of coherence and complexity (H4)

We employed a two-way analysis of variance (ANOVA) to investigate the interaction effects of coherence and complexity on participants’ physiological and psychological indicators. The results presented in Table 8 indicate no significant interaction effect for participants’ HR and RMSSD indicators. However, significant interaction effects were observed for both SCL (F = 4.62, P < 0.05, η² = 0.07) and PRS, indicating that coherence and complexity jointly influenced both physiological and perceived restoration outcomes. Further simple effects analysis, controlling for multiple factors at low levels, showed that the recovery value of SCL at low coherence (|M| = 2.26, SD = 1.03) was significantly higher than at high levels (|M| = 1.73, SD = 0.70), with F = 3.87, P < 0.05, ƞ^2 = 0.03. No significant differences were noted in SCL recovery values across coherence levels under medium complexity. Under high complexity, however, the SCL recovery value at low coherence (|M| = 1.79, SD = 0.61) was significantly lower than at medium (|M| = 2.44, SD = 0.68) and high (|M| = 2.26, SD = 0.74) coherence levels, with F = 6.11, P < 0.05, ƞ^2 = 0.05. No significant differences were noted across complexity levels at low coherence. At medium coherence level, the SCL recovery value in the low complexity condition (|M| = 1.99, SD = 0.74) was significantly higher than at high complexity (|M| = 2.20, SD = 0.62), F = 3.16, P < 0.05, ƞ^2 = 0.03. Under high coherence level, participants in the low complexity group showed significantly lower SCL perceived recovery values than those in the medium and high complexity groups (F = 4.62, P < 0.05, ƞ^2 = 0.04).

The main effect of the perceived restorativeness, as measured by the PRS, was found to be significant, as shown in Table 9. The complexity of the landscape exerted a strong significant influence on perceived restoration (F = 5.06, P < 0.001, ƞ^2 = 0.21), while coherence showed a moderate significant effect (F = 4.27, P < 0.05, ƞ^2 = 0.04). Delving deeper into a simple effects analysis, there was no notable difference in perceived restoration among college students across coherence levels at low complexity. At medium complexity, low coherence differed significantly from medium and high coherence, while perceived restoration effects for students were consistent between medium and high coherence landscapes. At high complexity, student perceived restoration in high coherence landscapes was notably higher than in low coherence landscapes, with no significant difference in perceived restoration in medium coherence scenarios when compared to low or high complexity scenarios. Under controlled low coherence factors, perceived restoration in low complexity scenarios was significantly lower than in medium and high complexity scenarios for students, with no significant recovery difference observed between medium and high complexity scenarios. The effects under controlled medium coherence were similar to those under low coherence. Lastly, under high coherence control, there were significant differences in perceived restoration among students across different environments of low, medium, and high levels of complexity, with perceived restoration increasing as complexity intensified.

## 4 Discussion

This study investigated how landscape coherence and complexity jointly shape psychophysiological stress recovery in a controlled VR experiment. Overall, vegetated courtyards produced better restorative outcomes than the non-vegetated control, particularly in SCL and PRS. Landscape complexity showed a positive association with perceived restoration, whereas coherence displayed a curvilinear relationship, with moderate coherence yielding the most favorable restorative responses. Most importantly, the interaction results indicated that the restorative benefits of higher complexity depended on the presence of sufficient coherence, suggesting that coherence plays a key moderating role in translating informational richness into restorative value.

### 4.1 Experiencing courtyard landscapes facilitates stress recovery in university students

Our one-way ANOVA comparing the non-vegetated built-courtyard control with the vegetated courtyard scenes showed significant differences in SCL and PRS, indicating better physiological and perceived recovery in the vegetated settings. By contrast, HR and RMSSD did not differ significantly between vegetated and control scenes. This pattern is consistent with evidence that brief exposures to nature are often more readily reflected in electrodermal and perceptual measures than in cardiac indices over short time windows. For example, Alvarsson et al. [40] found that natural sounds accelerated skin-conductance recovery after stress, while high-frequency HRV remained unchanged, and Brown et al. [17] reported that viewing nature scenes facilitated autonomic recovery after acute mental stress. Together, these findings suggest that vegetated courtyard environments can provide meaningful short-term restorative benefits for university students, especially at the level of perceived restoration and electrodermal recovery. This pattern is broadly consistent with prior restoration research showing that psychological and physiological indicators may not change in fully parallel ways across brief exposures [41], and that even relatively small doses of nature can generate measurable psychological benefits [42]. These findings support the value of incorporating accessible green courtyard spaces into campus settings.

### 4.2 The impact of courtyard landscape coherence on stress recovery in university students

This study showed that the restorative effects of courtyard landscapes varied with coherence in a curvilinear rather than linear manner. Moderate coherence produced the strongest psychological restoration, followed by high coherence, whereas low coherence performed worst. Specifically, PRS scores were significantly higher under moderate coherence than under low and high coherence, while HR, RMSSD, and SCL did not show significant main effects across coherence levels, although their mean values also tended to be most favorable at the moderate level. These findings indicate that coherence mainly influenced perceived restoration in the present short-term exposure, and they do not support Hypothesis H2 that higher coherence would lead to better recovery in a strictly linear way.

This pattern is broadly consistent with previous studies linking coherence to landscape preference and restoration [8,43]. By experimentally manipulating planting order and cluster regularity in VR, our study extends this literature by showing, under controlled conditions, that coherence influences restorative outcomes rather than preference alone. The results also align with Kaplan’s information-processing perspective, which emphasizes readability and comprehensibility in environmental experience [27,36].

A possible explanation is that moderately coherent landscapes balance legibility and naturalness more effectively than either low- or high-coherence settings. Compared with low coherence, moderate coherence reduces visual fragmentation and cognitive burden; compared with high coherence, it retains greater natural irregularity and may therefore feel less formal and more welcoming. This balance may explain why moderate coherence was more restorative than both extremes. Overall, the findings suggest that courtyard landscapes should not simply maximize order, but instead maintain an intermediate level of coherence that supports both comprehensibility and a natural appearance.

### 4.3 The impact of courtyard landscape complexity on stress recovery in university students

In this study, landscape complexity was manipulated quantitatively through entropy values (1, 2, and 4 bits). The results showed that complexity had a clearer effect on psychological restoration than on physiological recovery. PRS scores increased significantly with increasing complexity, whereas HR, RMSSD, and SCL did not show significant main effects, although some physiological means tended to improve as complexity increased. These findings partially support Hypothesis H3 and suggest that, within short-term courtyard exposure, complexity is more consistently reflected in perceived restoration than in autonomic recovery.

This result is consistent with previous studies reporting positive associations between landscape complexity, preference, and perceived restoration [7,44–46]. However, our study extends this literature in two important ways. First, rather than treating complexity as a general visual impression, we operationalized it quantitatively using Shannon entropy derived from plant-species composition. Second, by testing this relationship in a controlled VR stress-recovery experiment, we show that higher complexity predicts stronger perceived restoration even when short-term physiological responses remain less sensitive. This provides new empirical evidence that quantitatively defined landscape complexity is an important design variable for restorative courtyard environments.

A possible explanation is that more complex courtyard scenes provide richer informational content and greater “soft fascination,” thereby supporting the perceptual and attentional processes emphasized in Attention Restoration Theory. At the same time, short exposure duration may limit the extent to which this perceptual advantage translates into measurable autonomic change. Thus, our findings do not support a simple assumption that increasing complexity uniformly improves all dimensions of recovery. Instead, they suggest that complexity is an effective design lever primarily for enhancing perceived restoration, especially when integrated with other spatial attributes such as coherence.

### 4.4 Interaction of courtyard landscape coherence and complexity in stress recovery

The two-factor analysis showed that coherence and complexity did not act independently, but interacted in shaping stress recovery, particularly for SCL. Significant Coherence × Complexity interaction effects were observed for both SCL and PRS, whereas HR and RMSSD did not exhibit significant interaction effects. Notably, the interaction was theoretically most informative for SCL, while PRS also showed strong main effects of complexity and coherence. These findings indicate that the restorative effect of complexity is conditional rather than uniform: higher complexity does not automatically lead to better recovery, but depends on the degree of coherence in the scene. In this sense, the results support Hypothesis H4 and identify coherence as a key moderator of complexity-related restorative benefits.

This interaction is one of the central contributions of the present study. Previous re-search has generally examined coherence and complexity separately, or focused on their joint effects on landscape preference rather than restorative outcomes. By manipulating both variables simultaneously in a controlled VR stress-recovery experiment, this study provides new empirical evidence that coherence determines whether complexity is experienced as restorative or stressful. In other words, coherence functions as the condition under which informational richness can become a restorative resource rather than a perceptual burden. This moves the discussion beyond the simple assumption that more biodiversity or greater visual richness is always beneficial.

A plausible explanation is that coherence provides the perceptual structure required for complexity to be processed as engaging rather than chaotic. Under low coherence, in-creasing complexity may intensify fragmentation and reduce comprehensibility, thereby weakening restoration. Under higher coherence, however, the same increase in complexity can enrich the scene without overwhelming perception, allowing both the positive affective pathway proposed by Stress Reduction Theory and the attentional pathway proposed by Attention Restoration Theory to operate more effectively. Therefore, the interaction identified here suggests that restorative courtyard design should not treat complexity as an isolated target; instead, biodiversity and visual richness need to be supported by sufficient coherence to maximize restorative benefit.

### 4.5 Implications of the research findings

The findings of this study provide practical guidance for campus landscape design aimed at supporting student stress recovery, particularly in courtyards and other confined outdoor spaces associated with teaching buildings. First, compared with the non-vegetated control, vegetated courtyard settings produced better restorative outcomes, indicating that accessible green spaces should be prioritized in frequently used campus environments. This supports the inclusion of restorative planting in areas where students regularly experience academic pressure, such as teaching-building atriums, library surroundings, and spaces near dining facilities.

Second, the results suggest that restorative design should not focus on landscape complexity or coherence in isolation. Although greater complexity improved perceived restoration, its restorative value depended on sufficient coherence. In practical terms, this means that biodiverse and visually rich planting schemes should be accompanied by an appropriate degree of spatial order and legibility. Rather than simply increasing the number or variety of plants, designers should consider how planting composition, grouping, and structural organization work together to promote restoration. These findings provide a more specific design basis for creating campus courtyards that are both ecologically rich and psychologically restorative.

## 5 Conclusion

This study provides evidence that the design attributes of landscape coherence and complexity are important predictors of stress recovery in university students, with clear effects on perceived restoration and more limited, condition-dependent effects on physiology. Our primary findings revealed a nuanced picture: landscape complexity was positively and approximately linearly associated with higher PRS, whereas coherence was associated with better PRS in a monotonic pattern from low to moderate and then plateaued at high coherence (low <[moderate ≈ high]). Physiological main effects of coherence and complexity were not consistent across indices; however, a significant Coherence × Complexity interaction emerged for skin conductance level (SCL), indicating that physiological benefits materialize most clearly when higher complexity is paired with greater coherence. By contrast, PRS showed robust main effects of complexity and coherence, alongside a significant interaction effect, indicating that perceived restoration was shaped by both independent and joint influences of the two design attributes.

Collectively, these patterns do more than just codify a transferable design framework; they refine Stress Reduction Theory and Attention Restoration Theory. By theorizing coherence as a crucial moderator, our findings demonstrate how scene legibility acts as the condition that allows informational richness (complexity) to trigger parasympathetic rebound, thereby offering a synthesised theoretical pathway for restorative responses in designed environments. This framework, where entropy-based complexity and spatial coherence operate as coupled levers, supplies internationally relevant guidance for urban forestry practice. We anticipate that cross-national teams can apply the shared VR protocol and analytic pipeline to probe climatic or cultural moderators, accelerating the global evidence base on campus greening.

## 6 Limitations and future directions

This study has several limitations. First, the experiment included only three levels of coherence (scattered, formal, and clustered) and three levels of complexity (1, 2, and 4 bits), which limits the precision with which the relationships among these variables and stress recovery can be characterized. In particular, the observed positive association between complexity and restoration may not remain linear beyond the range tested here.

Second, the manipulated scenes focused primarily on vegetation characteristics, while other landscape elements such as topography, water features, paving, sky view, and architectural details were held constant. Although this control strengthened internal validity, it also reduced the ecological breadth of the experimental settings. Future studies could expand the framework by incorporating a wider range of landscape elements and combinations.

Third, the recovery period was limited to a 5-min exposure window, which may have been sufficient to detect changes in perceived restoration and electrodermal activity but less adequate for capturing more stable cardiac responses. In addition, HR and RMSSD were derived from PPG rather than ECG, which may have reduced sensitivity in detecting subtle autonomic changes.

Finally, the study was conducted with students from a single university, which may limit the generalizability of the findings across climatic, cultural, and institutional contexts. Future research should therefore test these relationships in more diverse campus environments, use longer exposure durations and more sensitive physiological measures, and explore the long-term restorative effects of courtyard landscapes.
